# Phenological responses to nitrogen and water addition are linked to plant growth patterns in a desert herbaceous community

**DOI:** 10.1002/ece3.4001

**Published:** 2018-04-25

**Authors:** Gang Huang, Chen‐hua Li, Yan Li

**Affiliations:** ^1^ State Key Lab of Desert and Oasis Ecology Xinjiang Institute of Ecology and Geography Chinese Academy of Sciences Urumqi Xinjiang China

**Keywords:** desert ecosystem, flowering, nitrogen deposition, plant phenology, precipitation increase, relative growth rate, spring ephemeral, spring‐summer annual

## Abstract

Increases in nitrogen (N) deposition and variation in precipitation have been occurring in temperate deserts; however, little information is available regarding plant phenological responses to environmental cues and their relationships with plant growth pattern in desert ecosystems. In this study, plant phenology and growth of six annuals in response to N and water addition were monitored throughout two consecutive growing seasons in 2011 and 2012 in a temperate desert in northwestern China. The effects of N and water addition on reproductive phenology differed among plant species. N and water addition consistently advanced the flowering onset time and fruiting time of four spring ephemerals; however, their effects on two spring‐summer annuals were inconsistent, with advances being noted in one species and delays in another. N and water addition alone increased plant height, relative growth rate, leaf number, flower number, and individual biomass, while their combinative effects on plant growth and reproductive phenology were dependent on species. Multiple regression analysis showed that flowering onset time was negatively correlated with relative growth rate of two species*,* and negatively correlated with maximum plant height of the other four species. Our study demonstrates that phenological responses to increasing precipitation and N deposition varied in annuals with different life histories, whereby the effects of climate change on plant growth rate were related to reproductive phenology. Desert annuals that were able to accelerate growth rate under increasing soil resource availability tended to advance their flowering onset time to escape drought later in the growing season. This study promotes our understanding of the responses of temperate desert annuals to increasing precipitation and N deposition in this desert.

## 1 INTRODUCTION

Phenology is the onset time and duration of biological events, and it determines species fitness and coexistence in plant communities (Forrest & Miller‐Rushing, 2010). Plant phenology is sensitive to variation in the ambient environment, and phenological shift as a result of species acclimation to environmental conditions (Meineri, Skarpaas, Spindelbock, Bargmann, & Vandvik, 2014; Parmesan & Yohe, 2003; Sparks & Carey, 1995) can trigger a series of changes in plant reproduction, community composition, and even ecosystem functions (Cleland, Chuine, & Menzel, 2007; Huang & Li, 2015; Schwartz, 2003). Plant phenological shift can affect competitive interactions among plants and the stability of trophic levels, thus consequently influencing ecosystem processes, including nutrient fluxes (Fogelstroem et al., 2017; Leverett, 2017).

Plant phenological responses to climate change are widely studied in terms of increasing temperature, varying precipitation, nitrogen (N) deposition, and elevated CO_2_ (Cleland, Chiariello, & Loarie, 2006; Gusewell, Furrer, Gehrig, & Pietragalla, 2017; Xia & Wan, 2013), of which altered precipitation patterns and N deposition are of particular interest for desert ecosystems.

Precipitation profoundly influences the timing and duration of the growing season, as well as plant nutrient acquisition in desert ecosystems (Aronson, Kigel, Shmida, & Klein, 1992; Bernal, Estiarte, & Peñuelas, 2011; Lasky, Uriarte, & Muscarella, 2016). Increasing atmospheric N deposition has been occurring on a global scale due to the combustion of fossil fuels and application of N fertilizer in agricultural ecosystems (Galloway et al., 2008). N deposition alters plant carbon assimilation, distribution, and growing season (Liu, Miao et al., 2017; Llorens & Penuelas, 2005), but the effects are species specific (Liu, Miao et al., 2017, Liu, Monaco et al., 2017; Peñuelas et al., 2004; Sherry, Zhou, & Gu, 2007; Xia & Wan, 2013; Zhang, Niu, Liu, Jia, & Du, 2014). For example, N addition advanced the flowering onset time and fruiting time of two spring‐summer annuals in the Chihuahuan Desert (Whitford & Gutierrez, 1989), as well as some forbs and a legume in a North American grassland, but had no impact on other plants (Cleland et al., 2006; Sherry et al., 2007).

Simultaneous variation in N and water availability can have synergistic effects on plant growth and phenology as decreased water availability might hamper N addition effects in arid and semiarid regions (Crimmins, Crimmins, & Bertelsen, 2010; Nord, Shea, & Lynch, 2011; Peñuelas et al., 2004; Sherry et al., 2007). Although different climate changes and environmental factors altered by anthropogenic activity, including precipitation and N, usually co‐occur, few studies have investigated the interactions of two or more factors (Liu, Miao et al., 2017, Liu, Monaco et al., 2017; Nord et al., 2011; Schuster & Dukes, 2017; Sherry et al., 2007). Therefore, manipulative field experiments are needed to investigate individual and combined effects of precipitation and N on plant growth and reproductive phenology in temperate deserts.

**Figure 1:**
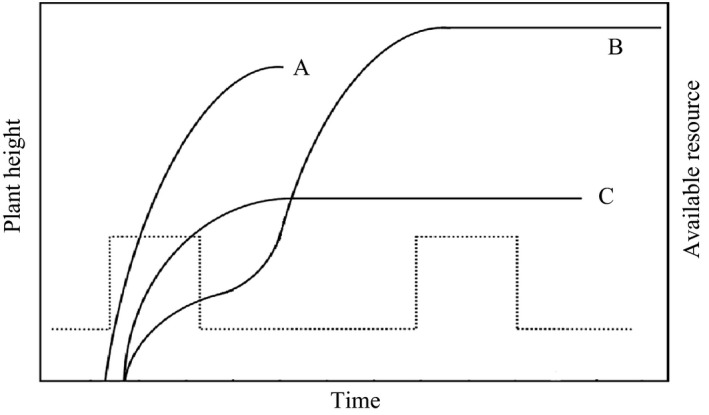
Schematic diagram showing three fundamental growth types for forbs in the desert. Drought‐escape and short life‐history ephemerals (Curve A); Drought resistance and long life‐history spring‐summer annuals (Curve B); Drought avoidance and long life‐history spring‐summer annuals (Curve C)

Plant responses to environmental changes can often be generalized by plant life‐history strategy. For example, annual grasses show earlier flowering onset time than forbs (Cleland et al., 2006), and early‐flowering species showed advanced flowering in response to warming, while late‐flowering species exhibited delayed flowering (Sherry et al., 2007). In general, plant species’ strategies in dry lands can be divided into drought avoidance and drought resistance, based on their water use. However, it is unclear how plant phenology responds to water or N availability in terms of life‐history strategy. There are three typical plant growth patterns in response to resource availability in desert ecosystems: Drought‐escape ephemerals only use soil resources at the first nutrient and water pulse in early spring; however, such a one bet strategy carries a risk of utter reproductive failure if resources are only available for a very short period (Figure 1curve A). Drought‐resistant spring‐summer annuals can endure drought during their reproductive period, and benefit from the second resource pulse in a low competition community because of the mortality of spring ephemerals (Curve B). Finally, drought avoiding spring‐summer annuals can survive drought periods through slow growth or aboveground dormancy and maintain reproduction in later resource pulses in autumn (Curve C). Accordingly, species with these distinct life‐history strategies may respond differently to water or N availability and thus modify the species coexistence in desert ecosystems (Rasmussen, Van Allen, & Rudolf, 2014; Zhang, Hu, & Zhang, 2016).

Given the importance of resource pulses for desert ecosystems, climate changes could have a profound effect on plant communities and ecosystem function. We investigated this using a manipulative experiment in a desert herbaceous community in northwestern China. Precipitation in northwestern China has been increasing over the past 50 years, and this trend is predicted to persist in the near future (Cholaw, Cubasch, & Lin, 2003; Ni & Zhang, 2000). Moreover, airborne N deposition increased from 1.3 g N m^−2^ year^−1^ in 1980 to 3.5 g N m^−2^ year^−1^ in 2012, and is expected to continue increasing primarily because of N fertilizer application to the encroaching farmland (Liu et al., 2013; Zhang, Zheng, & Liu, 2008). Hence, we examined the effects of simulated N deposition and increasing precipitation effects on reproductive phenology and growth of six dominant co‐occurring desert annuals throughout two consecutive growing seasons in 2011 and 2012. The six herbs can be grouped into spring ephemerals and spring‐summer annuals in terms of plant life‐history strategy. The specific aims of our study were to (1) assess whether N and water addition would alter plant growth patterns and phenophases, including flowering onset time, fruiting time, seed maturation time, and reproductive duration; (2) test whether plant phenological responses to N and water addition would be consistent among species or between two life‐history strategies (3) explore the role of plant growth traits in reproductive phenological responses, and determine how this is modified by water and N availability.

## 2 MATERIALS AND METHODS

### 2.1 Study site description

**Figure 2:**
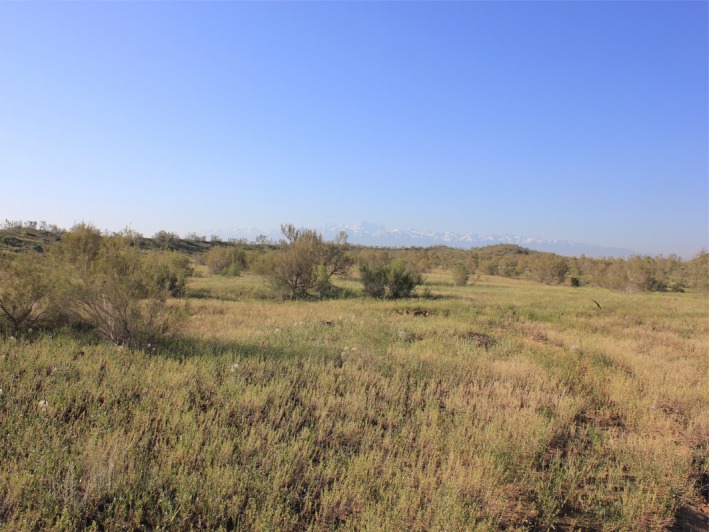
Typical shrub–forbs vegetation in the Gurbantunggut Desert, with lowlands between upper sand dunes dominated by *Haloxylon ammodendron and Haloxylon persicum, respectively,* and a dense, diverse herbaceous layer (*Erodium oxyrrhinchum, Alyssum linifolium*,* Leptaleum filifolium*,* Malcolmia africana, Salsola subcrassa*,* Ceratocarpus arenarius*)

The study was conducted at the Fu‐Kang Station of Desert Ecology (44°17′N, 87°56′E, 475 m a.s.l.) on the southern fringe of the Gurbantunggut Desert in northwestern China. The mean annual temperature is 6.6°C, and the average monthly temperature ranges from −19.4°C in January to 25.8°C in July. The mean annual precipitation from 1992 to 2012 is 160 mm, with c. 75% falling during the growing season from April to September. The topography of the Gurbantunggut Desert is characterized by south‐north longitudinal dunes with intervals of flat inter‐dune land. The vertical height from upland to lowland is 6–8 m, and the dune slope angle is c. 60°. The width of inter‐dune land is c. 200 m, and covered by well‐developed biological soil crusts (Su, Li, Cheng, Tan, & Jia, 2007; Su, Wu, Zhou, Liu, & Zhang, 2013). The soil of inter‐dune land is sandy loam and derived from alluvial deposits. The sandy dune soil is generally eolian. The vegetation comprises 35 species, in which 4 are shrubs, 5 are perennial herbaceous plants, and 26 are annuals (Huang & Li, 2015). The annuals can be grouped into spring ephemerals and spring‐summer annuals in terms of their life‐history strategy, with a coverage reaching 40% at peak plant growth (Figure 2). Spring ephemerals germinate immediately after snowmelt in mid‐late March, mature in mid‐May, and die off in late May–early June. Spring‐summer annuals usually exhibit low growth rates as accompanying species during the main growth period of spring ephemerals and show fast growth rates after the mortality of spring ephemerals.

### 2.2 Experimental set‐up

This study combined simulated N deposition and increased precipitation in a factorial design, resulting in four experimental treatments: N addition (N), water addition (W), combined N and water addition (NW), and controls (C) without extra water or N. The experimental set‐up was established in September 2010. The four treatments were randomly arranged in each of six blocks, making a total of 24 plots. The area of each plot was 10‐m × 10‐m, with a 10‐m buffer zone between adjacent plots. Based on the airborne N deposition of 3.5 g N m^−2^ year^−1^ registered in 2012 in northern China (He et al., 2007), 5 g N m^−2^ year^−1^ was selected as the simulated N deposition in our study; 1,667 g NH_4_NO_3_ was diluted in 15 L distilled water and evenly sprayed in each plot under N and NW treatments in early April 2011 and 2012. Simultaneously, 15 L distilled water was also added in each plot under C and W treatments. To simulate increased precipitation based on the predicted rate at our study site (Cholaw et al., 2003), an additional 15% precipitation was added in W and NW treatment plots after every rainfall event. Precipitation was 167.4 and 102 mm in 2011 and 2012, respectively, therefore, 25.1 and 15.3 mm precipitation was added in W and NW treatment in 2011 and 2012. Precipitation was collected using a 15 m^2^ “collection pan” installed beside each plot (15% of the plot area). The pan was erected with galvanized iron sheets at a slight angle of 2°, so that the collected rain could run into a plastic bucket buried in the soil. Collected rain was evenly sprayed onto the corresponding plot as soon as possible to prevent evaporation losses, usually during late afternoon or early morning after rainfall.

### 2.3 Environmental variable measurement

We measured air temperature, precipitation, soil moisture, and inorganic N content. Air temperature and precipitation were monitored hourly by an automatic meteorological station (Campbell Science Equipment, Logan, UT) at our study site. Soil moisture was monitored biweekly using time domain reflectometry in each plot at 0–20 cm depth (Diviner‐2000, Sentek Pty Ltd., Balmain, Australia). Soil water content reaches saturation immediately after snowmelt (volumetric water content 12%), and gradually decreases throughout May and June. When soil water content was below 5% and lasted >1 week, we defined this period of low soil moisture as a drought period (Figure S1). For soil inorganic N content, soil cores (5 cm in diameter and 5 cm in depth) were collected monthly in April‐September in each plot and composited to make one per plot. Samples were placed in plastic bags, transferred to the lab and stored at 4°C for further analysis. Soil dissolved inorganic N (DIN, including nitrate‐N (NO_3_^−^‐N) and ammonium‐N (NH_4_^+^‐N)) was extracted from 10‐g fresh soil with 50 ml 2 mol/L KCl. After shaking for 1 hr, DIN was filtered and the filtrate was measured using a continuous‐flow ion auto‐analyzer (AA3, Bran Luebbe, Germany).

### 2.4 Phenology and plant growth observations

The six species used for plant phenological observation accounted for more than 84% of the total coverage and 75.7% of the herbaceous biomass at our study site. The six species can be grouped into two plant life‐history strategies: spring ephemerals (*Alyssum linifolium*,* Leptaleum filifolium*,* Erodium oxyrrhinchum*and *Malcolmia africana*), and spring‐summer annuals (*Salsola subcrassa* and *Ceratocarpus arenarius*).

Flowering onset time, fruiting time, seed maturation time, and reproductive duration were monitored every 3 days from 28 March to the end of May and once a week thereafter in 2011 and 2012. At the beginning of each growing season, we tagged five mature individuals of each species in each plot at their first appearance. If a plant had more than one flower, we first recorded the current stage for each flower and then calculated a score by averaging all flowers. By this means we obtained a single “phenological score” for each individual. For the four spring ephemerals and *S. brachiata*, this included seven stages: pre‐flowering (stage 0), unopened buds (stage 1), open flowers (stage 2), old flowers (post‐anthesis; stage 3), initiated fruit (stage 4), expanding fruit (stage 5), and death of the aboveground parts with dehisced fruit (stage 6). For *C. arenarius* there were only four stages: pre‐flowering (stage 0), the presence of small axillary flowers and bract awns (flowering; stage 1), food bracts and long fruiting bract awns (seed production; stage 2), dried and yellow styles (seed maturation; stage 3). Phenological observation ended when all individuals had reached phenological stage 3 and 6 for *C. arenarius* and the other species, respectively). On each sampling date, the phenological scores for each species were averaged from the five individuals in each plot. The scoring of phenological stages was performed following the modified method described by Price and Waser (1998), Dunne, Harte, and Taylor (2003), and Sherry et al. (2007).

Plant height, leaf number, and flower number were also measured. In addition, individual biomass was calculated using the measured height and the allometric growth equation for each species from a previous study (Huang, Su, Zhu, & Li, 2016). Finally, plant density was obtained by recording the number of plants in a 5 m^2^ area in each plot at peak plant growth.

### 2.5 Statistical analysis

The Richards growth equation was used with the contraction‐expansion algorithm to describe the phenological data of each species within the plots (Richards, 1959):

$$Y\;=\;K/{\left(1\;+\;a\;\times \;e^{\left(-b\times X\right)}\right)}^{m}$$

where *Y* is the scored phenological stage, *K* is the maximum growth (measured during the last phenological stage), *a* is a parameter related to the first observation date, *b* is the growth rate (phenological stage change per day) over time *X* (days since the first observation date), and *m* is a parameter related to the curve shape. After utilizing the calibrated Richards equation for each species in each plot, flowering onset time (stage 1 for *C. arenarius* and stage 2 for the other herbs) and fruiting time (stage 2 for *C. arenarius* and stage 4 for the other herbs) were calculated for each individual plant in each plot. Because all herbs matured almost simultaneously, we used the observation date as the point of senescence. The reproductive duration was defined as the maturation time minus the flowering onset time. To explore the relationship between growth rate and flowering onset time, the relative growth rate before the flowering onset time was used to quantify plant growth rate. Relative growth rate was calculated using the following equation:

$$\text{RGR}\;=\;\left(\ln H_{2}\;-\;\ln H_{1}\right)/\left(t_{2}\;-\;t_{1}\right)$$

where *t*_1_ is the day at which the species first appeared) and *t*_2_ is the day at first flowering; and *H*_1_ and *H*_2_ are the height (in cm) of the plant at *t*_1_ and *t*_2_, respectively.

Initially, five‐way ANOVA was used to examine the effects of block, year, water, N, species, and their possible interactions on phenology. Water, N, and year were used as main fixed factors, species as a “split‐plot” factor and block as a random factor. Because there was no significant block effect, and significant species and year effects were observed, block was dropped from subsequent analyze. Repeated‐measures analysis of variance (RMANOVAs) was used to examine the main effects of N and water on plant phenological phases, maximum plant height and plant density of each species, with year as within‐subject factor and species, N and water as between‐subject factors. Because of the significant species × N and species × water interactions, we further used two‐way ANOVA to analyze the effects of N and water addition on each species in each year. Similarly, three‐way ANOVA was used to examine species and treatment effects on maximum plant height in each year. The multiple linear regressions with stepwise selection were used to examine the relationships of phenological phases and growth traits (maximum plant height, plant relative growth rate, leaf number, flower number, and individual biomass) for each species, and the autocorrelations between variables were diagnosed by VIF (the variance inflation factor) values and removed highly correlated predictors in equations according to correlation coefficient matrix. We quantified the effects of treatment on growth and phenophases using the RII index, calculated as:

$$\left(X_{c}-\;\;X_{\mathrm{treat}}\right)/\left(X_{c}\;+\;X_{\mathrm{treat}}\right)$$

where *X*_*c*_ and *X*_treat_ are the values of each variable in the control and treatment (N addition, water addition and N plus water addition), respectively (Armas, Ordiales, & Pugnaire, 2004). This index ranges from −1 to +1. Negative values indicate an increase in the variable considered (or a delay in the case of phenological events), while positive values indicate the opposite. Relationships between changes in growth and changes in reproductive phenological responses, as indicated by RII, were assessed using linear regression. The fitting of the calibrated Richards equation was performed using Matlab (Mathworks, Natick, MA), and the statistical analysis was performed using SPSS 13 (SPSS, Inc., Chicago, IL, USA).

## 3 RESULTS

### 3.1 Nitrogen and water addition effects on soil inorganic N content and soil moisture

N addition significantly increased soil inorganic N and nitrate‐N, while exerting no impacts on soil ammonium‐N (Table 1). N addition had no impacts on soil moisture (Table 1). Water addition slightly increased soil nitrate‐N and ammonium‐N (Table 1), and significantly increased soil moisture during the spring ephemeral growing period from April to June in both 2011 and 2012 (Table 1). Water addition significantly increased soil moisture at 0–10 cm by an average of 1.9% in 2011 and 2.9% in 2012 (Figure S1). Combined addition of N and water significantly increased soil inorganic N, but the accompanying increase in soil moisture was not significant (Table 1).

**Table 1:** Responses of soil inorganic nitrogen (IN), nitrate nitrogen (N‐NO_3_^−^), and ammonium nitrogen (N‐NH_4_^+^) contents as well as mean soil volumetric water content (SVWC) in control (C), nitrogen addition (N), water addition (W), and nitrogen plus water addition (NW) treatments during April to June (A–J) and July to October (J–O) in 2011 and 2012

Species	IN (mg/kg)	N‐NO_3_^−^ (mg/kg)	N‐NH_4_^+^ (mg/kg)	SVW(%) (2011A–J)	SVW(%) (2011J–O)	SVW(%) (2012A–J)	SVW(%) (2012J–O)
C	6.20 ± 0.31 a	5.14 ± 2.20 a	0.96 ± 0.20 a	5.17 ± 0.99 a	1.41 ± 0.18 a	2.36 ± 0.42 a	6.13 ± 0.83 a
N	14.34 ± 3.00 b	13.25 ± 2.91 b	1.09 ± 2.92 a	4.52 ± 0.90 a	1.26 ± 0.10 a	2.12 ± 0.31 a	6.15 ± 1.15 a
W	13.07 ± 1.84 ab	11.45 ± 1.87 ab	1.62 ± 0.46 a	7.54 ± 1.50 b	2.47 ± 0.31 a	4.13 ± 0.70 b	11.06 ± 1.79 a
NW	12.48 ± 2.09 b	11.55 ± 2.24 ab	0.93 ± 0.32 a	7.18 ± 1.27 ab	2.36 ± 0.36 a	3.51 ± 0.63 ab	10.06 ± 1.63 a

Data are shown as means ± *SE* for *n* = 6. Different lower‐case letters indicate statistically significant differences among treatments at *p *<* *.05.

### 3.2 Nitrogen and water addition effects on plant phenology

**Figure 3:**
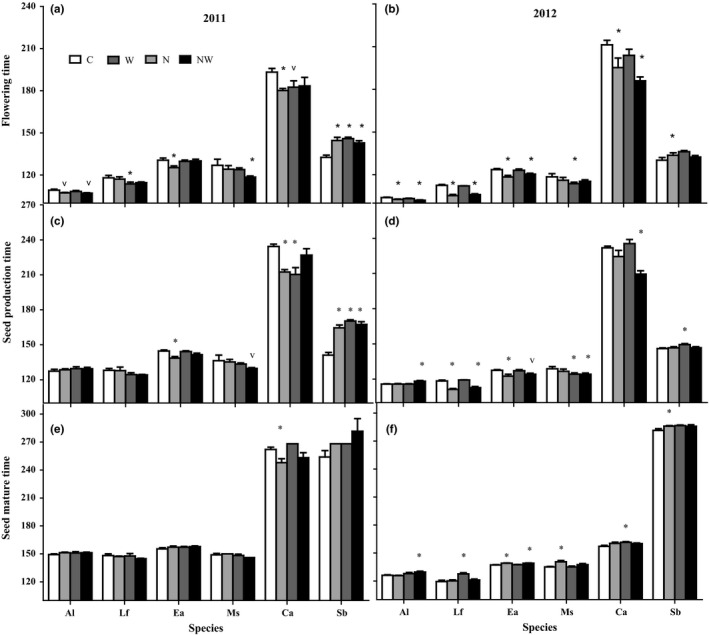
Mean day of four spring ephemerals (Al, *A. linifolium*; Lf, *L. filifolium*; Eo, *E. oxyrrhynchum*; Ms, *M. scorpioides*) and two spring‐summer annuals (Sb, *S. brachiata* and Ca, *C. arenarius*) flowering onset time (a and b), fruiting time (c and d) under control (C), nitrogen addition (N), water addition (W), and nitrogen plus water addition (NW) in 2011 and 2012. Bars indicate the mean ± 1 standard error, *n* = 6 for each bar. Species are listed in the order first flowering time observed in control plots, beginning in March with *A. linifolium* and ending in late October with *C. arenarius*. “*” and “v” on the error bar indicate significant difference between treatment and control (C) at *p* < .05 and *p* < .1 levels

N and water addition alone and in combination affected flowering onset time, but the effect varied by species (Table 2). Across two years, N addition significantly advanced the flowering onset time of the four spring ephemerals (*A. linifolium:* 1.4 days, *L. filifolium:* 5.6 days, *E. oxyrrhynchum:* 5.3 days and *S. brachiate*: 14.6 days; Figure 3), but delayed the flowering onset time of *C. arenarius* by 7.7 days. Water addition advanced the flowering onset time of *M. scorpioides* by 6.0 days (Figure 3), while delaying that of *C. arenarius* by 5.1 days (Figure 3). N and water addition together exerted significant interactive effects on flowering onset time of *C. arenarius* across the two years (Figure 3).

**Table 2:** The results (*F* values) of repeated measures ANOVAs on the effects of nitrogen addition (N), water addition (W), species (S), year (Y), and their interactions on flowering onset time (Flowering), fruiting time (Fruiting), seed maturation time (Maturation), and reproductive duration (Duration), maximum plant height (*H*_max_), relative growth rate (RGR), leaf number (LN) and species density (Density)

Items	Phenological traits				Growth traits			
	Flowering	Fruiting	Maturation	Duration	*H*_max_	RGR	LN	Density
N	15.81**	6.65*	0.10	7.40*	4.08*	0.73	6.34*	1.37
W	7.12*	0.02	7.65*	14.32**	8.31**	4.14*	13.07**	1.61
S	1626**	1953**	3567**	507.92**	69.52**	129.16**	27.56**	54.24**
Y	24.90**	6.39*	1021**	332.66**	132.63**	32.21**	7.61*	111.65**
N × W	0.02	0.26	1.25	0.92	3.63	3.01	1.31	1.39
W × S	6.33**	10.32**	3.05*	5.71**	1.75	2.72*	3.56*	0.61
N × S	5.25**	6.42**	6.65**	10.94**	2.01	2.11	4.01*	0.91
Y × W	1.84	4.27*	1.09	0.009	0.06	16.45**	4.65*	0.43
Y × N	1.59	5.53*	0.28	1.16	2.24	6.73*	2.93	0.93
Y × S	24.14**	42.28**	381.55**	128.43**	39.86**	54.59**	12.8**	56.62**
W × N × S	3.77*	6.12**	0.35	1.35	1.95	1.82	2.24	0.73
Y × W × N	0.14	2.72	0.004	0.03	5.73*	1.12	8.05*	0.71
Y × W × S	1.15	3.21*	2.03	0.18	2.25	6.00**	2.61*	1.02
Y × N × S	1.92	3.07*	5.16**	2.84*	2.07	0.65	4.28*	0.57
Y × W × N × S	1.29	14.27**	0.12	0.46	1.88	2.12	7.31**	0.77

*, and ** represent significant differences at *p *<* *.05 and *p *<* *.01, respectively, for *n* = 6 per treatment.

N addition significantly influenced fruiting time and interacted with species (Table 2). N addition significantly advanced fruiting time of the spring ephemerals of *L. filifolium* (5.4 days) and *E. oxyrrhynchum* (5.2 days), and spring‐summer annual of *C. arenarius* (13 days; Figure 3). Water addition had no impact on fruiting time across all six species (Table 2); however, it significantly advanced fruiting time of *M. scorpioides* (4 days), and delayed that of *S. brachiata* (11.6 days; Figure 3). N and water addition together had significant interactive effects on fruiting onset time of *C. arenarius* across the two years (Table 2).

N addition did not alter seed maturation time, when both growing seasons were analyzed (Table 2). However, N addition advanced seed maturation time of *C. arenarius* in 2011 and delayed that of *E. oxyrrhynchum*,* M. scorpioides,* and *S. brachiata* in 2012 (Figure 3). Water addition significantly affected seed maturation time and interacted with species (Table 2), with significant impacts on *C. arenarius* (Figure 3). N and water addition had no interactive effects on seed maturation time of any species (Table 2).

**Figure 4:**
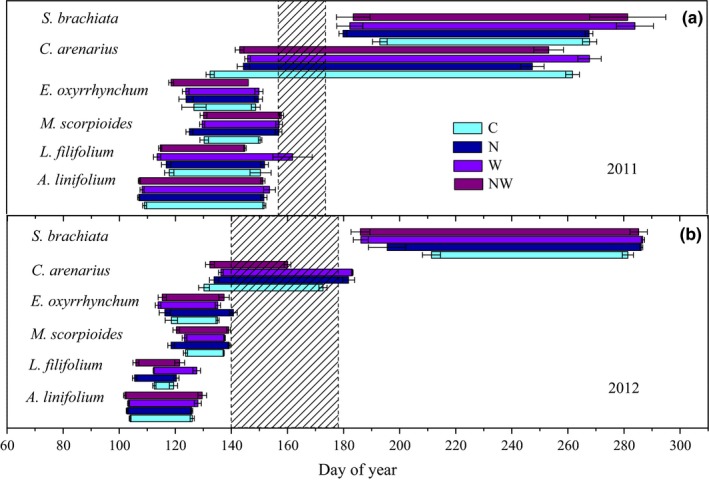
Timing and duration of the entire reproductive period composed of three phases (flowering, fruiting, and seed maturation) for the 6 herbs (*A. linifolium*,* L. filifolium*,* E. oxyrrhynchum*,* M. scorpioides*,* C. arenarius,* and *S. brachiata*) under control (C), nitrogen addition (N), water addition (W), and nitrogen plus water addition (NW) treatments in 2011 (a) and 2012 (b). The start and end points of the horizontal column indicate the average dates of flowering and seed maturation, respectively, and the length of the column indicates the reproductive duration. In each plot, the hatched columns indicate the drought periods in growing season. Data are presented as the mean ± *SE* of reproductive duration at the ends of both reproductive periods (*n* = 6)

Both N and water addition significantly altered reproductive duration (Table 2). N addition prolonged the reproductive duration of *E. oxyrrhynchum* and *S. brachiata* in both years, and prolonged that of *C. arenarius* in 2012 but shortened it in 2011 (Figure 4). Water addition prolonged the reproductive duration of *A. linifolium* and *S. brachiata* (Figure 4). N and water addition together had no interactive effects on reproductive duration (Table 2). The reproductive duration was significantly shorter in 2012 compared with that in 2011 for all species except *S. brachiata*, which showed no difference between the two years (Figure 4).

### 3.3 Nitrogen and water addition effects on plant growth

**Figure 5:**
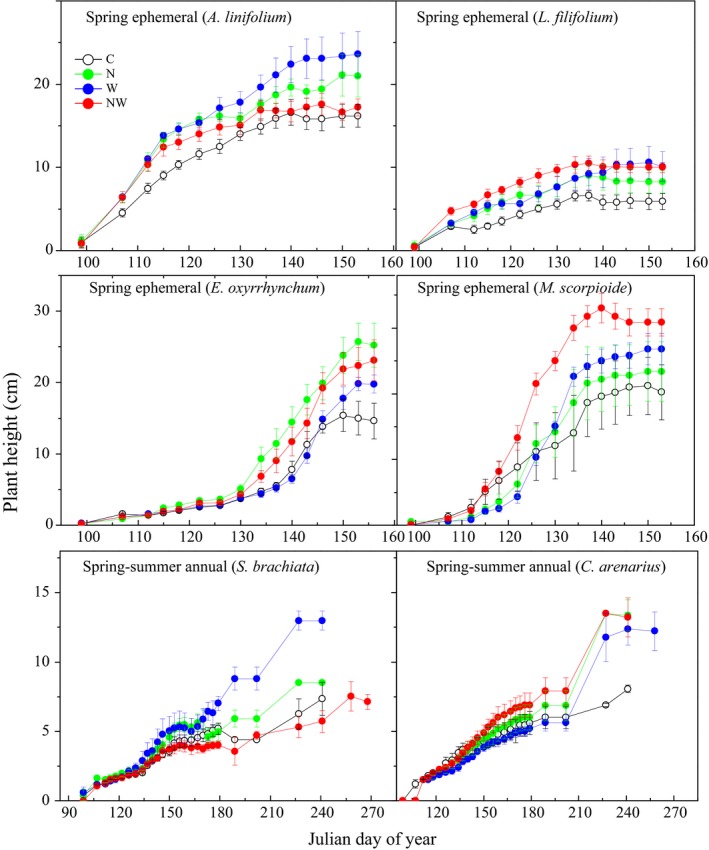
Plant height dynamics of four spring ephemerals (*A. linifolium*,* L. filifolium*,* E. oxyrrhynchum*, and *M. scorpioides*) and two spring‐summer annuals (*C. arenarius* and *S. brachiata*) under control (C), nitrogen addition (N), water addition (W), and nitrogen plus water addition (NW) treatments. Data are shown as the mean ± *SE* measured from April 10, 2011 at seed germination to September 26 when all the leaves of *S. brachiata* were shed (*n* = 18)

**Figure 6:**
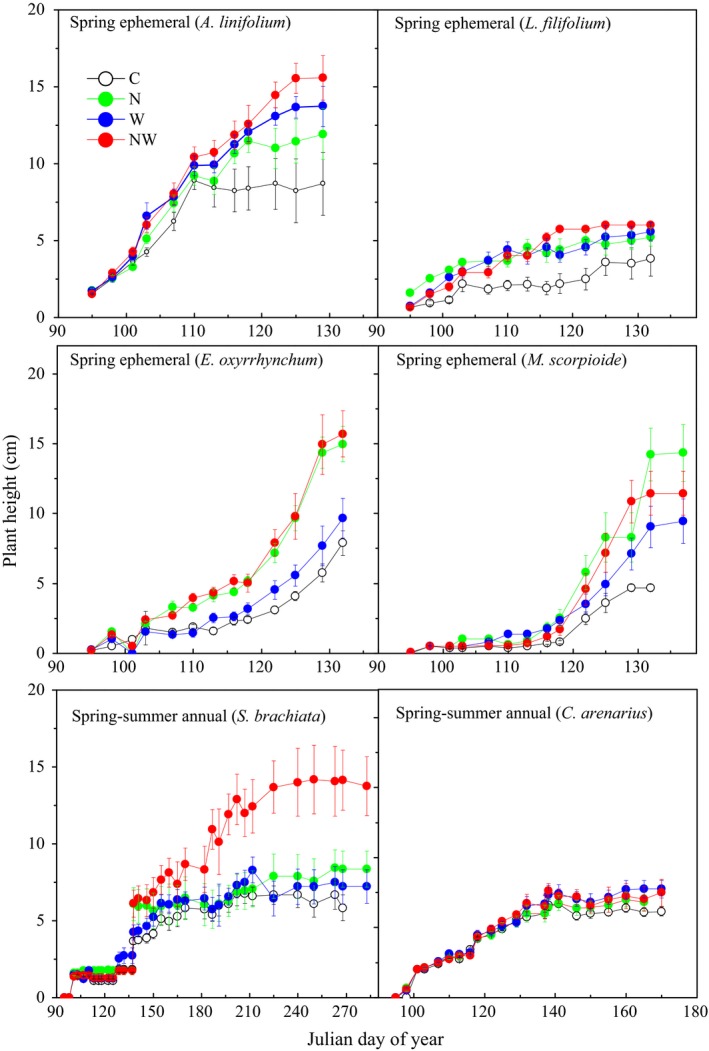
Plant height dynamics of four spring annuals (*A. linifolium*,* L. filifolium*,* E. oxyrrhynchum*, and *M. scorpioides*) and two spring‐summer annuals (*C. arenarius* and *S. brachiata*) under control (C), nitrogen addition (N), water addition (W), and nitrogen plus water addition (NW) treatments. Data are shown as the mean height ± *SE* measured from April 5, 2012, when seeds began germination to October 10 when all the leaves of *S. brachiata* were shed (*n* = 18)

N addition significantly promoted maximum plant height and leaf number (Table 2). N addition increased plant height of all species (Figures 5, 6). N addition had no impact on relative growth rate or species density, and only promoted leaf number of *E. oxyrrhynchum* and *S. brachiate* in 2011 and *E. oxyrrhynchum* in 2012 (Tables S1 and S2). Water addition promoted the maximum plant height of all species in two years (Table 2). Water addition also promoted plant relative growth rate and leaf number, but this varied by species and year (Table 2): water addition increased plant relative growth rate of *A. linifolium* and *S. brachiata* in 2011 and *M. scorpioides, C. arenarius* and *S. brachiata* in 2012 (Table S2). N and water addition had interactive effects on the relative growth rate of *A. linifolium* and *E. oxyrrhynchum* in 2011 and *A. linifolium*,* E. oxyrrhynchum*,* M. scorpioides* and *C. arenarius* in 2012 (Table 2; Table S2). The interactive effects of N and water addition on leaf number were dependent on year and species (Table 2), with significant effects on *L. filifolium*,* E. oxyrrhynchum* and *S. brachiate* in 2011 and *E. oxyrrhynchum* and *S. brachiate* in 2012 (Table S2).

### 3.4 Relationship between plant phenology and growth

**Figure 7:**
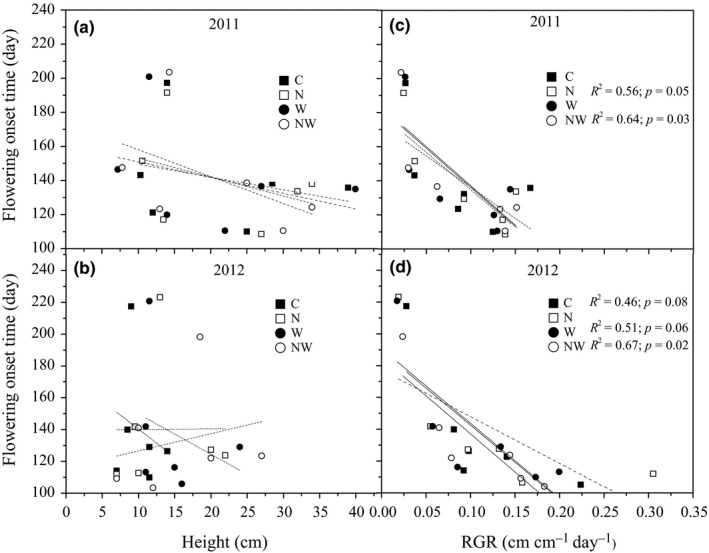
Bivariate relationships of flowering onset time with relative growth rate (A) and maximum plant height (B) under control (C), nitrogen addition (N), water addition (W), and nitrogen plus water addition (NW) treatments across all species in 2011 and 2012. Continuous solid lines indicate the significant linear correlations between flowering onset time and growth traits

Flowering onset time was negatively related to plant maximum height in all species except *A. linifolium* and *L. filifolium* (Table 3). Flowering onset time of spring ephemerals and fruiting time of *A. linifolium*,* E. oxyrrhynchum,* and *C. arenarius* were negatively related to plant relative growth rate (Table 3). Flowering onset time of spring‐summer annuals was negatively related with maximum height (Table 3). Flowering onset time and fruiting time of spring‐summer annuals were both negatively correlated with leaf and flower numbers (Table 3). There was no correlation between seed maturation time and plant growth traits except the correlation between seed maturation time and relative growth rate for *E. oxyrrhynchum, M. scorpioides,* and *C. arenarius* (Table 3). Reproductive duration was positively correlated with relative growth rate in *A. linifolium* and *M. scorpioides* and with plant maximum height in *C. arenarius* (Table 3). Relative growth rate explained 51% of the variation in flowering onset time and reproductive duration in *A. linifolium*, and 71% of variation in flowering onset time in *L. filifolium*. Plant maximum height explained more than 58% of the variation in flowering onset time for *E. oxyrrhynchum*,* M. scorpioides*,* S. brachiate,* and *C. arenarius* (Table 3). Plants with a faster relative growth rates in N addition and N plus water addition treatments showed significantly earlier flowering onset time in 2011 (Figure 7). Similar patterns occurred in water addition and N plus water addition in 2012 (Figure 7). In addition, the RII scores showed that the advanced flowering onset time was associated with the increased plant height under water addition in 2011, and with increased RGR under N addition in 2012 (Figure S2).

**Table 3:** The correlation coefficients of flowering onset time (FFD), fruiting time (FSD), seed maturation time (SMD), and reproductive duration (RD) with plant growth traits of maximum plant height (*H*_max_), maximum taproot length (RL_max_), relative growth rate (RGR), and leaf number per individual (LN), flower number per individual (FN), and biomass per individual, respectively

Source of variation	Plant growth traits						Stepwise regression equation
	*H*_max_	RL_max_	RGR	LN	FN	Biomass	
*A. linifolium*							
FFD	−0.43*	/	−0.51*	−0.17	−0.18	/	FFD = −35.61*RGR* + 107.56 *R* ^2^ = .51, *p *<* *.001 RD = 94.07*RGR* + 12.26 *R* ^2^ = .51, *p *<* *.001
FSD	−0.55*	/	−0.33*	0.26*	0.38*	/	
SMD	−0.07	/	−0.21	−0.03	0.05	/	
RD	0.21	/	0.32*	0.04	0.12	/	
*L. filifolium*							
FFD	−0.35*	/	−0.49*	−0.36	/	/	FFD = −82.14*RGR* + 126.24 *R* ^2^ = .71, *p *<* *.001
FSD	−0.17	/	−0.22	−0.08	/	/	
SMD	−0.07	/	−0.35	0.06	/	/	
RD	0.15	/	0.35	0.32	/	/	
*E. oxyrrhynchum*							
FFD	−0.65*	−0.55*	−0.65*	−0.48*	−0.66*	−0.27*	FFD = 131.2−0.4 *H*_max_ + 4.15biomass−0.95RL *R* ^2^ = .73, *p *<* *.001
FSD	−0.37*	−0.54*	−0.44*	−0.53*	−0.71*	−0.31*	
SMD	−0.46*	−0.44*	−0.49*	−0.38*	−0.38*	−0.31*	
RD	0.02	0.025	0.05	0.004	0.15	−0.08	
*M. scorpioides*							
FFD	−0.58*	/	−0.34	−0.42*	−0.21	/	FFD = −0.65 *H*_max_ + 123.35 *R* ^2^ = .58, *p *=* *.001
FSD	−0.46*	/	−0.13	−0.31	−0.28	/	
SMD	−0.02	/	−0.48*	−0.12	−0.26	/	
RD	0.35	/	0.54*	0.24	−0.02	/	
*S. brachiata*							
FFD	−0.67*	−0.37	−0.14	−0.56*	−0.58*	−0.35	FFD = −2.69 *H*_max_ + 222.8 *R* ^2^ = .69, *p *<* *.001
FSD	−0.62*	−0.42*	−0.03	−0.62*	−0.56*	−0.42*	
SMD	−0.67*	−0.29	−0.12	−0.56*	−0.50*	−0.29	
RD	0.14	0.18	0.10	0.10	0.25	0.18	
*C. arenarius*							
FFD	−0.65*	−0.08	−0.34	−0.43*	/	−0.09	FFD = −1.68 *H*_max_ + 146.53 *R* ^2^ = .65, *p *<* *.001 RD = 1.23 *H*_max_ + 20.29 *R* ^2^ = .45, *p *=* *.003
FSD	−0.54*	−0.08	−0.44*	−0.39*	/	−0.11	
SMD	−0.26	0.05	−0.46*	−0.25	/	−0.03	
RD	0.45*	0.10	0.03	0.24	/	0.09	

Stepwise regression analysis was further used to establish the relationships between phenology and growth traits. Asterisks indicate significant correlation between phenophases and growth traits at *p *<* *.05. / indicates no measurement was conducted.

## 4 DISCUSSION

### 4.1 Flowering and fruiting responses to nitrogen addition

N addition advanced the flowering onset time and fruiting time of all spring ephemerals and the spring‐summer annual of *S. brachiate*, while delaying those of the spring‐summer annual of *C. arenarius*. Species‐specific responses of reproductive phenology to N addition have also been reported in some previous studies. For instance, N addition advanced the flowering onset time and fruiting time of two spring‐summer annuals (*A. nuttallianus* and *C. stevioides*) in the Chihuahuan Desert (Whitford & Gutierrez, 1989), while delaying the flowering onset time of eight graminoid species in a Tibetan alpine meadow (Zhang et al., 2014). The species‐specific responses of reproductive phenology to N addition might be related to plant nutrient condition, spring‐summer annuals in our study have high arbuscular mycorrhizal fungi colonization (Chen, Shi, Tian, & Feng, 2008), and the extraradical hyphae of AM fungi can take up different forms of N from their surroundings and translocate N to the plant (Phillips, Brzostek, & Midgley, 2013). This may explain the high N absorption and the sensitive phenological responses (Phillips et al., 2013; Pregitzer, Burton, Zak, & Talhelm, 2008). The different phenological responses among species to N addition could have important ecological implications. For instance, the dramatic change in reproductive phenology can induce ecological asynchronies in plant community, thereafter affecting their pollinators or herbivores (Hegland, Nielsen, Lázaro, Bjerknes, & Totland, 2009; Inouye, 2008). Moreover, the differential responses among species to varying environmental cues can reflect and influence community resistance to disturbance (Stevens & Carson, 2001).

### 4.2 Flowering and fruiting responses to water addition

Our study showed distinct responses in flowering onset time to a 15% increase in precipitation and the magnitude of the responses was dependent on plant life‐history strategy. Spring ephemerals generally showed advanced flowering onset time by 0.3–5.2 days in our study, which falls within previously reported ranges (Cleland et al., 2006; Liu, Monaco et al., 2017; Meineri et al., 2014; Xia & Wan, 2012). However, two spring‐summer annuals showed opposite responses to water addition, with a delay in flowering onset of 5.9–13.3 days in *C. arenarius* but an advanced flowering onset of 5.9–10.7 days in *S. brachiate*. The higher response magnitude of spring‐summer annuals than spring ephemerals may be associated with the relatively long life‐span and reproductive duration of spring‐summer annuals in our study site (Valencia, Méndez, Saavedra, & Maestre, 2016).

The temporal gap in plant community phenology can generate additive trophic‐level influences by reducing the supply of vegetative resources for insects, such as pollen and seeds (Jamieson, Trowbridge, Raffa, & Lindroth, 2012). Due to the large variation of inter‐ and intra‐annual precipitation, reproductive phenology showed significant inter‐annual variation. In our study, reproductive phenological duration was markedly shorter in 2012 than in 2011, because 2012 was a much drier year, and thus, the overlap of reproductive stages was shortened between species. More importantly, *C. arenarius* represented a temporary connection in community phenology between early‐blooming spring ephemerals and the late‐blooming species such as *S. brachiata*: The reproductive duration of *C. arenarius* was 129.4 days in 2011 and 74.8 days in 2012, generating a 54.6‐day phenological gap in the herbaceous community in the control plots in 2012. Water addition advanced reproductive events of *S. brachiata* and prolonged those of *C. arenarius*, thereby closing the phenological gap in 2012. This result indicates that increasing precipitation can affect the phenology of herbaceous plants at the community level by expanding phenological duration and promoting reproductive phenology in this desert.

### 4.3 Flowering and fruiting responses to water plus nitrogen addition

Few studies have addressed the combined effects of N and water addition on reproductive phenology of desert annuals (Cleland et al., 2007; Gutierrez & Whitford, 1987; Liu, Monaco et al., 2017; Mauritz, Cleland, Merkley, & Lipson, 2014; Sharifi et al., 1988). Our study shows that the combination of N and water addition only advanced flowering onset time and fruiting time of *C. arenarius*. No interactive effects on spring ephemerals were noted for two reasons: First, water addition may have promoted soil N mineralization (Austin, Yahdjian, & Stark, 2004) or increased N leaching in the rhizosphere, as evidenced by the similar soil inorganic N content in water addition and control plots (Huang et al., 2016). Second, plant phenology is associated with the date of snowmelt (Philipp et al., 2016) because snowmelt in early spring usually equates to 40 mm rainfall, and has an overriding effects on seed germination of spring ephemerals in this desert (Fan, Tang, Wu, Ma, & Li, 2014). Further study is needed to test the interactive effects of snowmelt and N addition on the reproductive phenology of desert annuals.

Plant functional traits can be used to predict the community responses to climate changes. For example, N addition advanced flowering onset time of some forbs but delay that of grasses in North American grassland (Cleland et al., 2006). Similarly, the response magnitude of flowering onset time to the variation in precipitation varies among species (Cleland et al., 2006, 2007; Lasky et al., 2016; Prieto, Peñuelas, & Ogaya, 2008; Schwartz, 2003; Xia & Wan, 2012). In our study, although spring ephemerals consistently advanced flowering onset time and fruiting time in N and water addition treatments, the response magnitude varied among species and between years. Furthermore, the two spring‐summer annuals showed opposite phenological responses to treatments. Thus, our results suggest that spring ephemerals tend to accelerate plant growth and advance flowering onset time under favorable soil moisture and N, but plant life history cannot be necessarily used as a predictive proxy for phenological responses.

Plant relative growth rate can reflect plant survival strategies (Adler et al., 2014). Flowering is an indicator of switching from vegetative growth to reproductive growth, and plants usually prepare extensively for flowering via growth and mass storage (Schwartz, 2003). In our study, Flowering onset time was negatively correlated with the relative growth rate across six dominant species (Huxman, Barron‐Gafford, & Gerst, 2008; Kimball, Angert, & Huxman, 2011; Sun & Frelich, 2011), indicating that spring ephemerals tend to flower earlier than spring‐summer annuals. Given the phenological variation within a community, strategies adopted by spring‐summer annuals under frequent drought may be the crucial intrinsic mechanism that explains phenological responses to combined addition of water and N in deserts (Campanella & Bertiller, 2008; Dorji et al., 2013; Meineri et al., 2014; Sakkir, Shah, Cheruth, & Kabshawi, 2015). We thus deduced that drought‐escape species, like spring ephemerals, may accelerate plant growth and advance plant phenology under abundant soil moisture and N (Aronson et al., 1992; Fox, 1990; Kemp, 1983), while drought‐resistance species, like *C. arenarius*, might take more time to shift from vegetative growth to reproductive growth, thus showing high variation in response to climate changes.

The influence of plant growth traits on the responses of flowering onset time to N and water addition was associated with annual precipitation. In the dry year in 2012, plants exhibiting increased RGR showed advanced flowering onset time under N addition, whereas in 2011, water addition also increased RGR and advanced flowering onset time simultaneously. Our results partly indicated that plant growth changes could influence the flowering onset time in response to N or water addition, suggesting that plants can mitigate phenological changes by accelerating growth (Valladares et al., 2014).

## 5 CONCLUSION

Our study shows that N and water addition modified a set of plant phenological events and growth traits, and the response direction and magnitude of reproductive phenology are closely related to plant growth traits. Both N and water addition advanced the flowering onset time and fruiting time of spring ephemerals by promoting plant growth rate, although N and water had inconsistent effects on spring‐summer annuals. Additionally, N and water had no synergistic effects on plant phenology. The stimulatory effect of water addition on plant growth suggests a preliminary role of precipitation in shaping the composition and structure of a desert herbaceous community. More importantly, the advancement and expansion of reproductive phenology caused by increasing precipitation connected the gap of reproductive phenology at the community level during a dry year, which can buffer the negative effects of drought in the desert ecosystem. Thus, prolonged plant phenology and the promotion of plant growth and seed production in response to increasing precipitation will most likely benefit community composition and stability in the context of global climate change in temperate deserts.

## CONFLICT OF INTEREST

None declared.

## AUTHOR CONTRIBUTIONS

GH collected data and wrote the paper, CHL review the paper, YL designed the research.
