# Supplementary material for: Phenological responses to nitrogen and water addition are linked to plant growth patterns in a desert herbaceous community
Source: Ecol Evol. 2018 Apr 25;8(10):5139–52. doi: 10.1002/ece3.4001 (PMC5980538; doi:10.1002/ece3.4001)
Supplement: Supplementary file 1 [file ECE3-8-5139-s001.docx]

**Online Supplemental Materials**

**Table S1.** Species density (N m^-2^) and plant richness under the four treatments including control (C), nitrogen addition (N), water addition (W) and nitrogen plus water addition (NW) in 2011 and 2012 (mean ± SE, n=6). Different letters indicate significant difference between treatments at *P* < 0.05.

| Species/Richness | LH | Herbaceous density in 2011 (N m^-2^) | | | |  | Herbaceous density in 2012 (N m^-2^) | | | |
| --- | --- | --- | --- | --- | --- | --- | --- | --- | --- | --- |
|  |  | C | N | W | NW |  | C | N | W | NW |
| *Erodium oxyrrhynchum* | E | 53±8 a | 51±5a | 67±8 b | 64±3 b |  | 12±1.1 a | 11±3.5 a | 13±1 a | 18±1.3 b |
| *Alyssum linifolium* | E | 7±3 a | 6.3±3 a | 13±3 b | 12±4 b |  | 15±3 a | 22±6.2 ab | 20±3 ab | 26±5 b |
| *Malcolmia africana* | E | 6±1 a | 8.2± a | 6±1 a | 13±5 a |  | 1±0.6 a | 2.2±0.9 a | 2.1±0.5 a | 3±1 a |
| *Leptaleum filifolium* | E | 18±12 a | 14.6±3 a | 12±3 a | 19±6 a |  | 0.2±0.2 a | 0.3±0.1 a | 0.5±0.2 a | 0.4±0.2 a |
| *Schismus arabicus* | E | 30±17 a | 82±19 b | 45±23 c | 42±20 c |  | 8±4 a | 80±25 c | 27±10 b | 48±17 c |
| *Tragopogon ruber* | P | 0.1±0 a | 0.2±0.1 a | 0.4±0.2 a | 0.16±0.1 a |  | 0.2±0.2 a | 0.5±0.3 a | 1±0 a | 0.6±0.3 a |
| *Nonea caspica* | E | 2.7±1 a | 0.2±0.1 a | 0.1±0 a | 0a |  | 5±0 a | 0.2±0.1 a | 3±1 a | 3.5±1 a |
| *Alyssum desertorum* | E | 1.3±1 a | 0a | 0 a | 0.04±0 a |  | 1.3±1 a | 0.8±0.1 a | 0 a | 0.7±0.2 a |
| *Amberboa turanica* | E | 1.9±0.6 a | 2.4± a | 3.3±0.8 a | 0.04±0.0 a |  | 1.8±0.8 a | 3.8±1.5b | 4.5±1.5 b | 4.2±1 b |
| *Arnebia guttata* | E | 0 a | 0 a | 0 a | 0.04±0 a |  | 0 | 0 | 0 a | 0 a |
| *Nepeta micrantha* | E | 0.3±0.1 a | 0.04±0 a | 0 a | 0.04±0 a |  | 0a | 0a | 0.8±0.2 a | 0 a |
| *Ceratocarpus arenarius* | A | 8±7 a | 1.8±0.5 b | 12±5.9 ac | 12±4 c |  | 16±1 a | 13.8±4 a | 21±3 b | 55±8 c |
| *Salsola subcrassa* | A | 146±95 a | 77±19 b | 177±38 c | 173±48 ac |  | 1.8±0.4 a | 5.8±3.1 a | 0 a | 0.6±0.2 a |
| *Tetracme recurvata* | E | 0a | 0.3±0.1a | 0 a | 0 a |  | 0.4±0.2 a | 1.5±0.8 a | 0.9±0.2 a | 0.8±0.2 a |
| *Haloxylon ammodendron* | P | 2.6±1.9 a | 0.9±0.4 a | 4±1.3 a | 1.6±0.6 a |  | 0.8±0.5 a | 2.2±0.7 a | 0.8±0.2 a | 0.8±0.2 a |
| *Limonium bicolor* | P | 0.1±0 a | 0.04±0 a | 0.16±0 a | 0.04±0 a |  | 0.8±0.6 a | 1.3±0.4 a | 0.4±0 a | 0.5±0 a |
| *Salsola passerina* | S | 8.4±6 a | 1.6±1 a | 5.9±4.9 a | 4.2±2.5 a |  | 12±2 a | 5.3±2 a | 4±1 b | 1±0.2 b |
| *Descurainia sophia* | E | 0.1±0 a | 0 a | 0 a | 0.04±0 a |  | 0 a | 0 a | 0 a | 0 a |
| *Chenopodium glaucum* | A | 0.1±0 a | 0.16±0 a | 0 a | 0 a |  | 0 a | 1.5±0.8 a | 0 a | 0 a |
| *Euphorbia turczaninowii* | A | 0 a | 0 a | 0 a | 0 a |  | 0.4±0.1 a | 0a | 0 a | 0.16±0 a |
| *Salsola ruthenica* | A | 0.1±0 a | 0.08±0 a | 0.04±0 a | 0.04±0 a |  | 0 a | 0.8±0.6 a | 0 a | 0.5±0 a |
| *Carex physodes* | P | 0 a | 0 a | 1.2±1 a | 0 a |  | 0 a | 0 a | 0 a | 0.8±0 a |
| *Lactuca undulate* | A | 0.1±0 a | 0.04±0 a | 0.08±0 a | 0.08±0 a |  | 0 a | 0 a | 0 a | 0 a |
| *Hyalea pulchella* | A | 0.4±0.1 a | 0.7±0.5 a | 0.48±0.2 a | 0.2±0.1 a |  | 0.4±0 a | 1.4±0.5 a | 0.2± 0 | 1.5±0.5 a |
| *Seriphidiam santolinum* | P | 0.4±0.2 a | 0.04±0 a | 0.12±0 a | 0.2±0.1 a |  | 0 a | 0.5±0.3 a | 0.5± 0 | 0.2±0 a |
| *Koelpina linearis* | P | 0.1±0 a | 0.04±0 a | 0 a | 0 a |  | 0 a | 0.2±0.1 a | 0.8±0 a | 0 a |
|  |  |  |  |  |  |  |  |  |  |  |
| *Richness* |  | 12±0.3 a | 13.4±1a | 13.4±1 a | 14±1 a |  | 8.8±0.6 a | 12.5±0.5 a | 10±0.6 a | 11.8±0.7 a |

LH, A, P and E indicates life history, annual, perennial and ephemeral plants.

Table S2. Growth traits of the six annuals (*A. linifolium, L. filifolium, E. oxyrrhynchum, M. scorpioides, C. arenarius,* and *S. brachiata*) under control (C), nitrogen addition (N), water addition (W) and water plus nitrogen addition (NW) treatments during the growing season of 2011. RGR and LN indicate the relative growth rate and leaf number per individual, respectively. * and ^ indicate significant differences at *P* < 0.05 and marginally significant differences at 0.05 < *P* < 0.1 between control and treatment.

| Source of variation | 2011 | |  | 2012 | |
| --- | --- | --- | --- | --- | --- |
|  | RGR(cm·cm^-1^·day^-1^) | LN (No.) |  | RGR(cm·cm^-1^·day^-1^) | LN(No.) |
| *A. linifolium* | | | | | |
| C | 0.05±0.007 | 54.4±20 |  | 0.12±0.007 | 23.8±3.1 |
| N | 0.08±0.003* | 76.2±14.8 |  | 0.12±0.008 | 25.6±2.7 |
| W | 0.12±0.007* | 82.2±46.4 |  | 0.14±0.008 | 18.5±1.5 |
| NW | 0.12±0.007* | 30.34±13.5 |  | 0.15±0.005* | 31.0±3.6^ |
| *L. filifolium* | | | | | |
| C | 0.08±0.008 | 29.4±3.8 |  | 0.12±0.02 | 22±6.3 |
| N | 0.1±0.01 | 47.4±10.2 |  | 0.10±0.01 | 36±9.6 |
| W | 0.11±0.01 | 47.4±10.2 |  | 0.15±0.01 | 39.4±11.0* |
| NW | 0.11±0.02 | 59.8±13.1* |  | 0.12±0.01 | 25.5±2.7 |
| *E. oxyrrhynchum* | | | | | |
| C | 0.038±0.006 | 6.6±0.4 |  | 0.10±0.002 | 8.5±0.3 |
| N | 0.075±0.013* | 13.8±2.8* |  | 0.12±0.002* | 15.2±1.4* |
| W | 0.053±0.009 | 8.0±0.2 |  | 0.10±0.004 | 9.6±1.32 |
| NW | 0.063±0.020* | 12.4±2.0* |  | 0.12±0.004* | 20.2±1.8* |
| *M. scorpioides* | | | | | |
| C | 0.097±0.014 | 17.4±3.4 |  | 0.08±0.005 | 6.7±0.5 |
| N | 0.129±0.004* | 26.8±8.1 |  | 0.08±0.003 | 14±2.6 |
| W | 0.124±0.006 | 38.8±6.9* |  | 0.059±0.003* | 7±0.6 |
| NW | 0.125±0.012^ | 32.8±1.9 |  | 0.06±0.002* | 20.8±5.77^ |
| *C. arenarius* | | | | | |
| C | 0.027±0.002 | 42.4±7.4 |  | 0.067±0.002 | 78.8±10.6 |
| N | 0.027±0.004 | 43.7±8.4 |  | 0.046±0.002* | 102.4±25.4 |
| W | 0.025±0.001 | 28±2.3 |  | 0.050±0.002* | 182.8±55.6^ |
| NW | 0.031±0.002 | 50.8±8.1 |  | 0.053±0.002* | 205.2±40.1 |
| *S. brachiata* | | | | | |
| C | 0.02±0.001 | 35.8±9.6 |  | 0.017±0.003 | 110.3±36.4 |
| N | 0.020±0.001 | 93±3.2* |  | 0.009±0.002* | 97.1±35.1 |
| W | 0.024±0.001* | 118.6±20.6* |  | 0.012±0.001^ | 71.2±17.24 |
| NW | 0.018±0.001 | 81.3±9.0* |  | 0.015±0.001 | 332.0±73.9* |

Figure S1. Daily average air temperature, daily precipitation (A) and soil volumetric water content (SWC) (mean ± SE) (%) under the four treatments including control (C), nitrogen addition (N), water addition (W) and nitrogen plus water addition (NW) at the study site.

Figure S2. Relationships between the effect size of water (W), nitrogen (N) and nitrogen plus water addition (NW) on growth (height and relative growth rate (RGR) ) and flowering onset time, as indicated by RII (Armas et al. 2004) in 2011 and 2012. Al, *A. linifolium*; Lf, *L. filifolium*; Eo, *E. oxyrrhynchum*; Ms, *M. scorpioides*; Sb, *S. brachiate*; Ca, *C. arenarius*)
